# Differences in perceived threat and trauma in children during the COVID-19 pandemic

**DOI:** 10.1186/s13034-023-00628-5

**Published:** 2023-06-29

**Authors:** Gabriele Kohlboeck, Anna Wenter, Kathrin Sevecke, Silvia Exenberger

**Affiliations:** 1 Department of Child and Adolescent Psychiatry, Psychotherapy and Psychosomatics, Tirol Kliniken, Milser Straße 10, 6060 Hall in Tirol, Innsbruck, Austria; 2 Department of Child and Adolescent Psychiatry, Medical University of Innsbruck, Innsbruck, Austria; 3 Department of Psychology, University of Innsbruck, Innsbruck, Austria

**Keywords:** Children, Trauma, Stress, Mental health, COVID-19, Psychopathology, PTSD

## Abstract

**Background:**

The past 2 years of the COVID-19 pandemic were stressful for most children and adolescents; some children may have experienced a high level of stress and trauma. To date, no study has examined self-reported stress and trauma levels due to COVID-19 in children. This study aimed to assess perceived threat, exposure and trauma symptoms in children aged 7–13 years. In addition, we explored whether parent-reported factors could predict a higher risk of COVID-19 vulnerability in their children.

**Method:**

Cross-sectional data were collected from 752 children to assess COVID-19 threat, exposure and trauma symptoms using the self- and parent-reported Child and Adolescent Trauma Screening Self-Report (CATS) Trauma questionnaire. We used exploratory analyses (factor analysis of mixed data and hierarchical clustering) to identify subgroups (i.e., clusters) of children sharing similar characteristics in the dataset. Linear regression modeling was applied to determine the likelihood of higher threat and vulnerability in children with parent-reported COVID-19 threat, exposure, CATS trauma symptoms, behaviors on the Child Behavior Checklist (CBCL), and posttraumatic growth (PTG).

**Results:**

We identified a high-risk group of children reporting clinically relevant trauma symptoms and COVID-19-related fears. Parents’ reports of trauma could be used to identify children at high risk.

**Conclusions:**

Approximately 25% of children reported moderate to clinically relevant levels of trauma symptom. It is especially important to offer adequate support to these children to ease the trauma and prevent their symptoms from developing into psychopathology.

## Introduction

Children are most vulnerable to events that create stress and fear. The past 2 years of the COVID-19 pandemic were stressful for most children and adolescents due to social distancing, wearing masks, school closures, disrupted peer relationships, COVID-19 infections, loss of loved ones, and a general sense of unpredictability in their lives [11]. These events may have been interpreted as very threatening to some children [60] and can set off stress activation that subsequently causes physiological and behavioral responses [33, 54] similar to the symptoms of posttraumatic stress disorder (PTSD) [10]. Concerns about the COVID-19 pandemic, anxiety and fear of contracting the virus, public health instructions, and measures for confinement and social and physical distancing may be traumatic events [40].

Children are very heterogenous with respect to how they perceive and judge stressful events. Although the pandemic may have changed all children’s lives, some children have a higher risk of experiencing the harmful effects of this pandemic. Therefore, it is important to identify children who are at a higher risk for experiencing a high level of trauma to stop the process from further developing into psychopathology.

Research on trauma symptoms during the COVID-19 pandemic has almost exclusively focused on adults [28], to date, there is only one study available based on parent-reported traumatic symptoms in children [31]. The COVID-19 pandemic may disproportionately affect children, as children often suddenly lose essential resilience factors, namely, the support of parents, friends, neighbors and the social infrastructure that is normally in place to ensure their safety and provide assistance [13].

There is a considerable need to obtain knowledge about children experiencing trauma by using children’s self-reports. Clinicians and other practitioners rely mainly on symptoms of mental health reported by parents, teachers or other caregivers rather than by children’s self-reports [3, 4, 41]. In pediatrics, there is a growing sense that the voice of children and adolescents should be given greater importance, particularly with regard to their mental health [4, 16, 22]. Research has demonstrated that 6-year-old children have adequate understanding and produce reliable and valid self-reports of their health, these reports become stronger after age 7 in general populations [46]. However, no study has identified different risk groups based on children’s self-reports in the general population.

Therefore, the goal of this study was to examine differences in perceived threat and trauma in children during the COVID-19 pandemic. We hypothesize that there is variance in children’s reactions to the COVID-19 pandemic. In addition, we aimed to explore whether trauma symptoms, psychopathology, COVID-19-related worries, financial and job problems and posttraumatic growth (PTG) reported by parents can predict a higher risk of COVID-19-associated problems among children.

## Material and methods

### Setting and procedure

This study is part of the Tyrolean COVID-19 Children’s Study in North Tyrol (Austria) and South Tyrol (Italy) and aimed to investigate the impact of the COVID-19 pandemic on 3- to 13-year-olds, taking the perspective of children, parents, and educators into account, and was performed between March 2020 and July 2022. The Tyrolean COVID-19 Children’s Study is an online study using the software LimeSurvey (LimeSurvey GmbH, 2021) and CHES (ESD, 2020). Parents were recruited in schools and by advertisements through North and South Tyrolean media (print media as well as online formats) to encourage broad participation. The study included five measurement time points (March/April 2020, December 2020/January 2021, June/July 2021, December 2021/January 2022, and June/July 2022). The eligibility criteria were living in North or South Tyrol, parenthood of a 3- to 13-year-old child, proficiency in the German language, and the cognitive ability to fill out an online questionnaire.

### Study population

In this substudy, only data from self-reports of children aged between seven and 13 years were considered. Of the 868 children from the Tyrolean COVID-19 Children’s Study whose parents consented to the assessment, complete data were available for 752 participants (50.8% girls). Parent-reported data included 466 parent–child dyads.

For the study, approval by the local Ethics Committee (Medical University of Innsbruck [No. 1183/2020]) and written consent from the participant’s families were obtained.

### Study design

For the Tyrolean COVID-19 Children’s study, a repeated cross-sectional study design (RCS) was used. At each of the five time points of measurement, a new sample of participants was included. Repeated cross-sectional (RCS) design is also known as a “pseudo-longitudinal” design [62] and is used for analyzing populations or group changes over time [44]. Hence, the results cannot be used to examine individual changes but rather changes over time at a group level [44].

### Measures

#### Assessment of trauma

Children and their parents completed the German version of the 7- to 17-year-old children/youth Child and Adolescent Trauma Screening Self-Report (CATS 7-17), [47] directly based on the DSM-5 criteria for posttraumatic stress disorder (PTSD).

The English, German, and Norwegian versions of the CATS trauma screening questionnaire have excellent reliability, with Cronbach’s α coefficients ranging between 0.88 and 0.94 in clinical samples of youth suffering from single and multiple traumatic events. The convergent-discriminant validity pattern showed medium to strong correlations (*r* = 0.40–0.82) with measures of depression and anxiety [47]. Posttraumatic stress symptoms were measured by 20 items rated on a rating scale with the following anchors: 0 = “Never”, 1 = “Once in a while”, 2 = “Half the time” and 3 = “Almost always”. A total symptom score was calculated by summing the raw scores of Items 1–20. For the present analysis, the CATS subscale criteria B (intrusions), C (avoidance), D (negative alterations in cognitions and mood) and E (hyperarousal) were used and scored according to the German CATS version [47]. CATS raw scores < 21 can be interpreted as normal (0–15) or moderate trauma-related distress (15–20), whereas scores ≥ 21 indicate a clinically relevant level of symptoms, which could meet the criteria for probable PTSD.

#### Pandemic exposure

Children and their parents provided information on their degree of exposure by answering four yes/no questions: the child themselves had COVID-19; a family member had COVID-19; a family member was hospitalized with COVID-19; and a family member died from COVID-19. For the total score of pandemic exposure, all events were counted and then weighted according to the severity of the possible burden of the event: occurrence of child infected with COVID-19 weighted by 10, occurrence of parent infected with COVID-19 weighted by 25, occurrence of family member hospitalized weighted by 50, occurrence of family member’s death weighted by 100. The weights on the occurrence of COVID-19 infections (10 vs. 25) are based on findings of the Co-SPACE project reporting that children are more concerned about friends and family getting sick than about catching the virus themselves [12] or more worried about transmitting the virus to their grandparents [23].

#### Threat experience

Children and parents reported the experienced threat caused by COVID-19 through four yes/no questions: worry that a family member could become ill; worry that the children could themselves become ill; worry that a family member could die; and worry that the children themselves could die. These four items were summed into a total score of threat experience.

#### Financial and job problems

Financial and job problems of the family related to the coronavirus crisis were reported by children and parents using two yes/no items (“Have you and your family experienced financial problems due to the coronavirus crisis?”, “Has your mom or your dad lost their jobs due to the coronavirus crisis?”).

#### Child behavior checklist (CBCL)

Parents completed the German version of the CBCL 6–18 years; they were asked to assess their child’s behavior for internalizing problems (Emotionally Reactive, Anxious/Depressed, Somatic Complaints, Withdrawn, Sleep Problems) on a 3-point Likert scale for each item (0 = not true; 1 = somewhat or sometimes true; 2 = very true or often true). In addition, the aggressive behavior scale was included, which is part of the externalizing symptom scale of the CBCL.

#### Chronic illness and psychological treatment before the COVID-19 pandemic

Children also reported whether they had a chronic illness or were undergoing psychological treatment before the coronavirus crisis using two yes/no items.

Background variables included information about the child’s nationality, age, and gender.

#### Posttraumatic growth (PTG)

Posttraumatic growth (PTG) is defined as positive changes resulting from an individual’s struggle with traumatic or stressful events [7]. PTG was measured using the open-ended question (“What positive impact do you think the coronavirus crisis has/had on your child?”). If parents’ responses indicated one or more positive impacts, PTG was scored as 1 (“yes”), if parents stated that they did not notice any positive impact, PTG was scored as 0 (“no”).

### Statistical analysis

Only participants with complete datasets for the continuous and categorical variables were included in the analysis. We used exploratory data analysis methods to identify subgroups (i.e., clusters) of children sharing similar characteristics in the dataset. We first performed factor analysis of mixed data (FAMD), a principal component (PCA) method for analyzing quantitative and qualitative variables, to reduce the dimensions of the data into few components containing the most important information in the data. FAMD was performed using the R package FactoMineR (https://cran.r-project.org/package=FactoMineR), and the factoextra package (https://cran.r-project.org/package=factoextra) was used to extract the FAMD results. The first 13 components explained > 80% of the total variance, and all components were retained for further cluster analysis. The function HCPC in the FactoMineR package was used to compute hierarchical clustering on principal components.

To compare child-reported and parent-reported differences across clusters, we used one-way analysis of variance (ANOVA) for continuous variables and used the Pearson chi-squared test to assess differences between categorical variables; means and standard deviations (SD) were used to describe continuous variables. To investigate how children’s trauma and exposure scores changed over time during the pandemic, a two-way ANOVA was conducted using the R package CGP function with the factor “time” (five samples drawn at the different time points) and the factor “cluster” (0 = “No-Risk”, 1 = “High-Risk”). The CATS trauma total score and the exposure were modeled separately as the dependent variables.

To assess whether parent-reported data can predict a higher risk of COVID-19 vulnerability in children, we used logistic regression models with parent-reported data as predictors (independent variables) and the cluster solution resulting from hierarchical clustering as the outcome (dependent variable: 0 = “No-Risk”, 1 = “High-Risk” Cluster). Only one predictor at a time was entered into a logistic regression model adjusted for age, gender and nationality. A 0.05 level of significance was set.

The statistical software package R Studio (R Studio version 2022.07.2) was used for FAMD, hierarchical clustering on principal components, and two-way ANOVA., SPSS version 27.0.0 2019 was used for ANOVA and logistic regressions.

## Results

### Factor analysis of mixed data and hierarchical cluster analysis

**Fig. 1:**
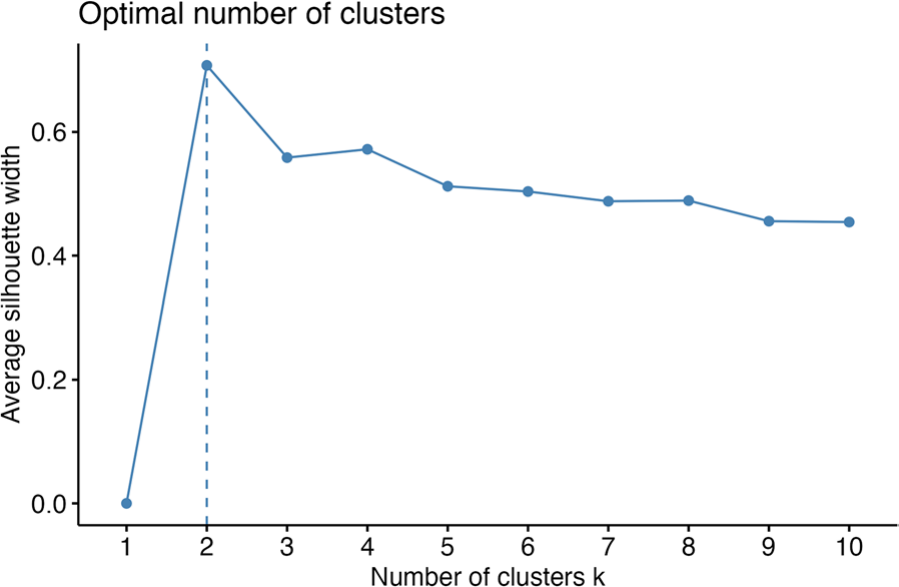
Optimal numbers clusters

**Fig. 2:**
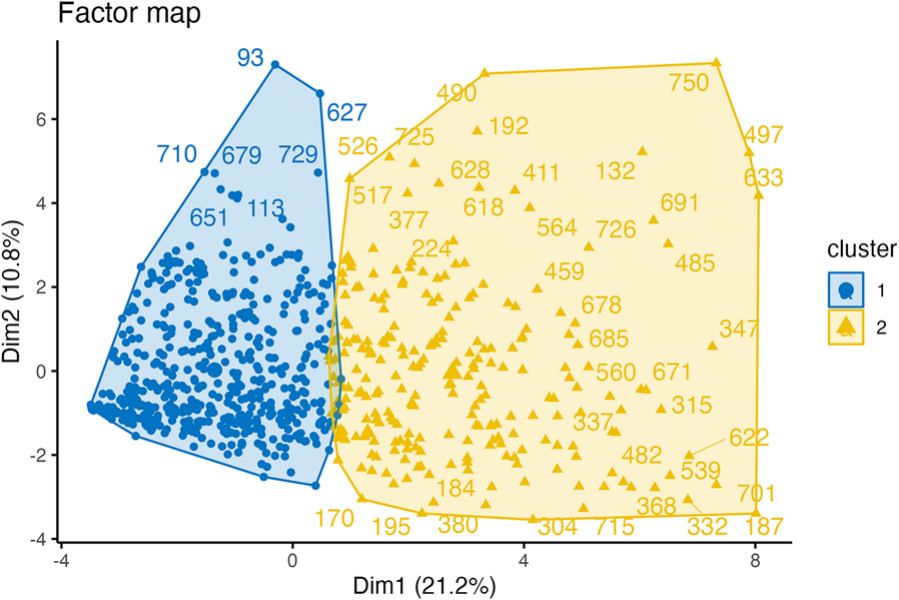
2Cluster_Solution

Hierarchical cluster analysis favored a two-cluster solution to describe children with different traumas, exposures, and fears due to the COVID-19 pandemic (Fig. 1). From both a statistical point of view as well as from child characteristics shown between clusters, the two-cluster solution proved to be ideal for explaining the results of child reports: the two clusters are as different from each other as possible, and a minimum overlap between clusters (marked by different colors) can be seen (Fig. 2). When the results were divided into three clusters, more members in cluster 1 and cluster 2 tended to overlap than in the 2-cluster solution.

However, in the three-cluster solution, there were disproportionally more members in cluster 2 measured at later time points (Dec 2021, June 2022), when COVID-19 exposure was at its highest and most children and family members had been sick with COVID-19. With a four-cluster solution, clusters were found to overlap even more.

As this high-exposure cluster at later time points had similar characteristics of threat and trauma to cluster 1 (“No-Risk”), a 2-cluster solution was finally chosen.

### Children’s characteristics of the 2-cluster solution

A total of n = 752 children aged between seven and 13 years (mean age was 9.89, SD 1.69) were included in this study. The results of differences in children of the 2-cluster solution are presented in Table 1.

**Table 1:** Children self-reported differences in background, COVID-19-related worry and exposure, and CATS trauma among the two clusters

Variable	“No-risk” N = 505		“High-risk” N = 247		*χ*^2^	F
	*M* *N*	SD %	M N	SD %		
Gender (girls)	251	49.7	131	53.0	0.74	
Age	9.81	1.65	10.07	1.75		4.18*
Nationality: Southern Tyrol	227	45.0	81	32.8	10.14**	
Time point of measurements (waves) with five different samples drawn					26.0***	
March 2020	140	70.7	58	29.3		
December 2020	133	58.8	93	41.2		
June 2021	71	61.7	44	38.3		
December 2021	51	63.0	30	37.0		
June 2022	110	83.3	22	16.7		
Psychological treatment before COVID-19 (yes)	4	0.8	31	12.6	51.68***	
Chronic Illness before COVID-19 (yes)	16	3.2	16	6.5	4.46*	
Fear of getting COVID-19 (yes)	87	17.2	172	69.6	201.77***	
Fear someone in family is getting COVID-19 (yes)	224	44.4	220	89.1	137.13***	
Fear of dying of COVID-19 (yes)	9	1.8	105	42.5	213.93***	
Fear someone in family is dying of COVID-19 (yes)	135	26.7	211	85.4	230.02***	
Fear total score	0.23	0.26	0.72	0.28		572.33***
Child ha COVID-19 (yes)	106	21.0	57	23.1	0.43	
Someone in family had COVID-19 (yes)	208	41.2	131	53.0	9.41**	
Someone in family was in hospital due to COVID-19 (yes)	17	3.4	31	12.6	23.41***	
Someone in family died due to COVID-19 (yes)	4	0.8	11	4.5	11.38**	
Exposure total score	14.87	22.36	26.30	35.57		28.83⁎⁎⁎
Financial problems in family due to COVID-19 (yes)	52	10.3	66	26.7	33.82***	
Mom or dad have no work parents (yes)	30	5.9	42	17.0	23.45***	
CATS trauma total score	6.69	5.13	21.75	10.09		736.06***
Normal (0–15)	460	91.0	63	26.0	363.32***	
Moderate (15–20)	36	7.0	56	23.0		
Clinically relevant (≥ 21)	9	2.0	128	52.0		
CATS re-experiencing trauma	1.44	1.50	5.56	3.36		539.26***
CATS avoidance	0.47	0.92	2.42	1.67		427.06***
CATS negative emotions	2.05	2.25	6.72	4.19		395.13***
CATS arousal	2.73	2.36	7.04	3.28		423.50***

Percentages with different letters are significantly different at *p* < 0.05

⁎*p* < 0.05

⁎⁎*p* < 0.01

⁎⁎⁎*p* < 0.001

In short, the first cluster, “No-Risk” (n = 505 [67.2%]), describes children with low levels of worry, trauma and exposure (Table 1). The second cluster, “High-Risk” (n = 247 [32.8%]), describes children reporting not only high levels of trauma, exposure and threat about COVID-19 but also a higher likelihood of financial and job problems in the family.

The distribution of boys and girls in the two clusters was approximately the same (see Table 1). The distribution of nationality was significantly different, with a higher proportion (45%) of children from Southern Tyrol, i.e., Italy, in the first cluster (“No-Risk”), and a lower proportion from Southern Tyrol in the cluster “High-Risk” (32.8%).

**Fig. 3:**
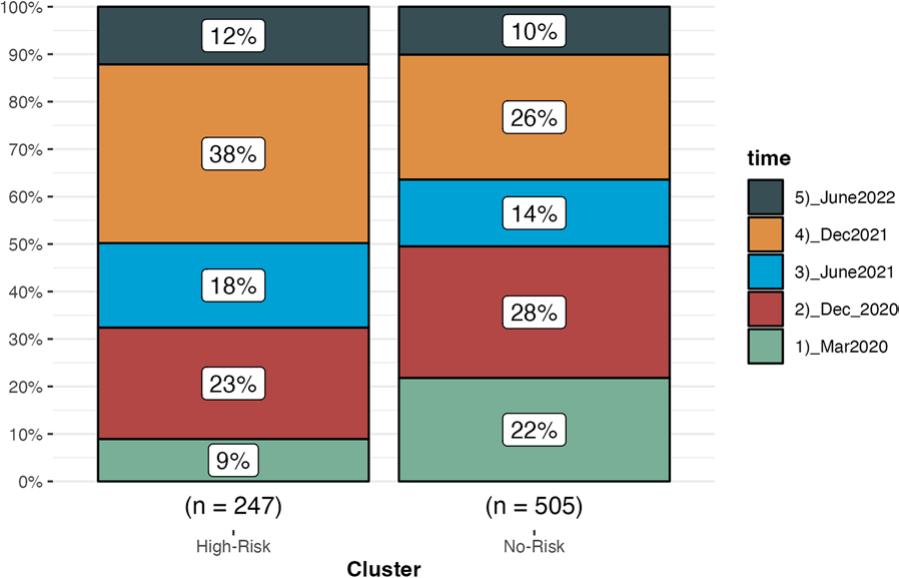
Time points by clusters

Proportions of time points of measurements *within* clusters show that children in the “No-Risk” cluster were most likely to be assessed in March 2020 [27.7%], December 2020 [26.3%], and June 2022 [21.8%]) but less likely to be assessed in December 2021 (10.1%) (Fig. 3). Children in the “High-Risk” cluster were most likely to be assessed in December 2020 (37.7%) and less likely to be assessed in June 2022 (8.9%).

As shown in Table 1, children in cluster 2 (“High-Risk”) were significantly older (mean age 10.07, SD 1.75) than the children from cluster 1 (“No-Risk” [mean age 9.81, SD 1.65 years]). Children in the “High-Risk” cluster also reported more frequently having been in psychological treatment or having suffered from chronic illness before the COVID-19 pandemic than those in the “No-Risk” cluster. Children reported that the perceived threat related to COVID-19 was significantly higher in the “High-Risk” cluster than in the “No-Risk” cluster. Regarding COVID-19 exposure weighted by severity of burden, we found a significantly higher exposure total score in the “High-Risk” cluster than in the “No-Risk” cluster. Although children in the “High-Risk” cluster reported having been sick with COVID-19 with approximately the same frequency as in the “No-Risk” cluster, the number of sicknesses or hospitalizations of a member of their family was significantly higher in that cluster.

The children in the “High-Risk” cluster reported a mean CATS total score of 21.8 (SD 10.1), indicating a clinically relevant level of trauma symptoms (cutoff > 21 [56]), whereas CATS total mean scores were approximately 3 times lower in the “No-Risk” cluster (mean 6.70, SD 5.13).

Children in the “High-Risk” cluster also experienced significantly higher CATS criteria B (intrusions), criteria C (avoidance), criteria D (negative alterations in cognitions and mood) and criteria E (hyperarousal) symptoms than those in the “No-Risk” cluster. Children in the “High-Risk” cluster also reported significantly higher levels of COVID-19 threat total scores than those in the “No-Risk” cluster.

Compared to the “No-Risk” cluster, parents of children in the “High-Risk” cluster also reported having more money problems due to COVID-19 (26.7% vs. 10.3%). The results further showed that the parents of children in the “High-Risk” cluster were more likely to have lost their jobs due to COVID-19 (17.0% vs. 5.9%).

### Changes in child-reported trauma, threat and exposure over time at the group level

**Fig. 4:**
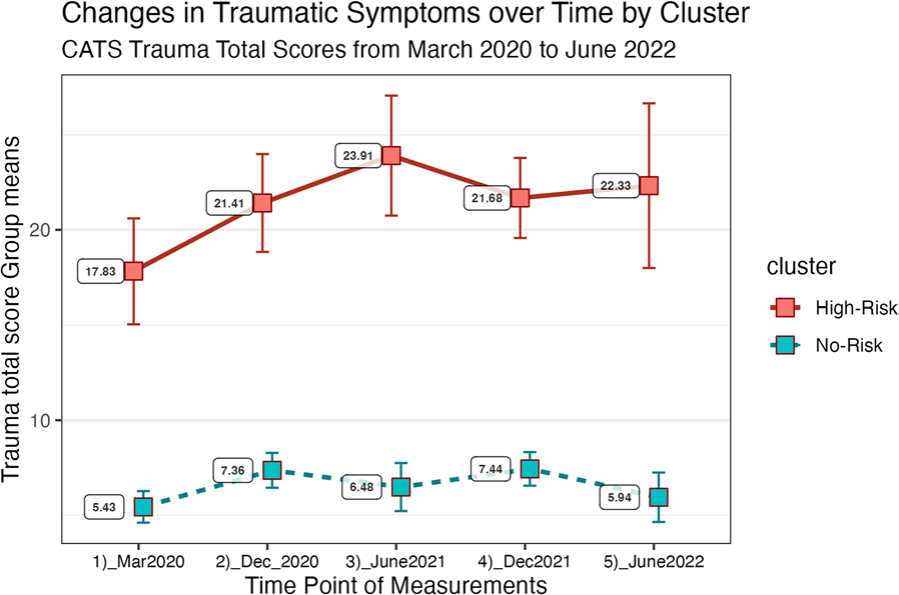
Changes in traumatic symptoms over time by cluster

Time (p 0.036) and cluster (p < 0.001) had significant effects on changes in total trauma scores, while the time * cluster interaction did not have a significant effect. When examining the overall change in trauma scores for the two clusters over time (5 different samples measured at the five time points), levels of trauma were high within the “High-Risk” cluster from March 2020 to June 2021 (see Fig. 4), and they were somewhat lower in December 2021 and June 2022. For the “No-Risk” cluster, levels of trauma were particularly higher between March 2020 and December 2020 and were lower in June 2021 and June 2022.

Time and cluster had significant effects on exposure (p < 0.001), while the time * cluster did not have a significant effect. Exposure levels for the “High-Risk” cluster were particularly high (mean 24.8, SD 44.9) at the start of the pandemic (March 2020) compared to the “No-Risk” cluster (mean 10.91, SD 10.9). However, exposure levels continued to increase over time until June 2022 in both the “High-Risk” cluster (mean 41.0, SD 33.7) and the “No-Risk” cluster (mean 33.4, SD 16).

Threat scores remained relatively stable over time in both clusters (i.e., time was not a significant factor), with 2.5 times higher threat levels in the “High-Risk” cluster than in the “No-Risk” cluster (p < 0.001 [data not shown here]).

### Differences in parent-reported trauma, psychopathology, worry, and financial and job problems

The results in Table 2 show that parent responses matched with the child responses for all items (CATS trauma, CBCL psychopathology, COVID-19-related threat and exposure) with the exception of actual exposure to someone in the family who died due to COVID-19. Children reported a significantly higher frequency of family member deaths than their parents.

**Table 2:** Parent-reported differences in background COVID-related worry, exposure and PTG between the two clusters

Variable	No risk		High-risk		*χ*^2^_(4)_	F
	*M* *(N)*	SD %	*M* *N*	SD %		
Threat of getting COVID-19 parents (yes), N = 403	68	24.5	81	64.3	58.69***	
Threat someone in family is getting COVID-19 parents (yes), N = 403	134	48.2	109	87.2	39.19***	
Threat of dying of COVID-19 parents (yes), N = 399	19	6.6	37	32.7	45.73***	
Threat someone in family is dying of COVID-19 parents (yes), N = 384	60	22.2	85	74.6	93.43***	
Child had COVID-19 parents (yes), N = 442	62	20.1	33	24.8	0.27	
Someone in family had COVID-19 parents (yes), N = 442	132	42.7	79	59.4	10.37**	
Someone in family was in hospital due to COVID-19 parents (yes), N = 442	10	3.2	12	9.0	6.58*	
Someone in family died due to COVID-19 parents (yes), N = 442	6	1.9	6	4.5	2.32	
Exposure total score parents, N = 430	113.56	17.34	121.14	22.53		14.21***
Our family had to borrow money from the bank parents (yes), N = 292	2	1.0	7	7.7	9.41**	
Our family has less money to buy things parents (yes), N = 292	24	11.9	13	14.3	0.31	
Mom or dad have changed work parents (yes), N = 292	15	7.5	12	13.2	2.45	
Mom or dad have no work parents (yes), N = 292	18	9.0	13	14.3	1.88	
Alcohol problem in family parents (yes), N = 291	2	1.0	1	1.1	0.01	
CATS trauma total score parents N = 418	4.97	5.84	13.56	10.49		114.53***
CATS re-experiencing trauma parents, N = 418	0.87	1.54	2.82	2.85		81.70***
CATS avoidance parents, N = 418	0.36	0.93	1.18	1.40		50.68***
CATS negative emotions parents, N = 418	1.61	2.53	4.54	4.24		77.43***
CATS arousal parents, N = 418	2.13	2.25	5.01	3.57		99.53***
CBCL total score parents, N = 437	26.55	25.04	56.45	36.16		99.36***
CBCL anxious depressed parents, N = 437	2.58	3.18	6.48	5.33		89.90***
CBCL withdrawn depressed parents, N = 437	1.48	2.24	4.54	3.05		62.38***
CBCL somatic complaints parents, N = 437	1.26	3.32	2.21	3.42		56.49***
CBCL attention problems parents, N = 437	3.32	3.37	6.59	4.40		71.72***
CBCL social problems parents, N = 437	3.29	5.85	7.61	8.69		36.86***
CBCL aggressive behavior parents, N = 437	4.79	4.93	8.71	6.69		46.83***
CBCL internalizing parents, N = 437	5.32	6.52	13.35	10.30		96.34***
CBCL externalizing parents, N = 437	9.31	9.59	16.94	12.99		46.83***
Post-traumatic growth (PTG) parents, N = 359	197	81.1	83	71.6		4.14*

Means with different letters are significantly different at *p* < 0.05

⁎*p* < 0.05

⁎⁎*p* < 0.01

⁎⁎⁎*p* < 0.001

With regard to differences between the two clusters, parents of “High-Risk” children reported higher mean scores of threat, exposure, trauma and psychopathology (CBCL) than parents of the “No-Risk” children. Finally, parents reported a significantly lower Posttraumatic Growth (PTG) score for “High-Risk” children than for “No-Risk” children (Table 2).

The results from adjusted logistic regression models showed (Table 3) that the likelihood of being in the “High-Risk” cluster was significantly associated with parent-reported CATS trauma total scores and all CATS subscales. The relationship was also significant for the CBCL total score and all CBCL subscales.

**Table 3:** Adjusted odds ratios (OR) for the risk of being in the “high risk” cluster according to the parent-reported family background, COVID-19 threat, exposure, CATS trauma, CBCL scales, and post-traumatic growth (PTG)

Parent-reported variables	Risk of being in the “high risk” cluster				
	Nagelkerk’sche R^2^	OR	95% CI		p
Threat of the child to get COVID-19 (n = 402)	0.21	4.93	3.09	7.86	< 0.001
Threat someone in the family gets COVID-19 (n = 402)	0.23	7.56	4.11	13.52	< 0.001
Threat of dying of COVID-19 (n = 398)	0.18	6.31	3.38	11.76	< 0.001
Threat someone in family will die due to COVID-19 (n = 383)	0.33	9.60	5.71	16.12	< 0.001
Child was COVID-19 sick, N = 441	0.05	1.13	0.70	1.86	0.635
Someone in family had COVID-19, N = 441	0.07	1.73	1.13	2.65	0.011
Someone in family was in hospital due to COVID-19, N = 441	0.07	2.84	1.16	6.99	0.023
Someone in family died due to COVID-19, N = 441	0.07	4.53	1.34	15.37	0.015
Exposure total score, N = 429	0.10	1.02	1.00	1.03	0.007
Alcohol problems in the family (n = 290)	0.05	0.88	0.07	10.92	0.923
Family had to borrow money from the bank (n = 291)	0.09	8.78	1.73	44.56	0.009
Family had less money to buy things (n = 291)	0.06	1.25	0.59	2.63	0.561
Mom or Dad had no job any more (n = 291)	0.06	1.75	0.80	3.84	0.161
CATS trauma total score (n = 417)	0.30	1.14	1.10	1.17	< 0.001
CATS re-experiencing (n = 417)	0.24	1.52	1.35	1.71	< 0.001
CATS avoidance (n = 417)	0.17	1.81	1.49	2.20	< 0.001
CATS negative emotions (n = 417)	0.23	1.29	1.20	1.38	< 0.001
CATS arousal (n = 417)	0.27	1.39	1.28	1.51	< 0.001
CBCL internalizing (n = 436)	0.26	1.12	1.09	1.15	< 0.001
CBCL anxious/depressed (n = 436)	0.25	1.24	1.17	1.32	< 0.001
CBCL withdrawn/depressed (n = 436)	0.19	1.33	1.22	1.45	< 0.001
CBCL somatic complaints (n = 436)	0.18	1.31	1.19	1.43	< 0.001
CBCL social problems (n = 436)	0.15	1.01	1.06	1.12	< 0.001
CBCL attention problems (n = 436)	0.21	1.22	1.15	1.30	< 0.001
CBCL aggressive behavior (n = 436)	0.16	1.12	1.08	1.16	< 0.001
CBCL total score (n = 436)	0.26	1.03	1.02	1.04	< 0.001
Post-traumatic growth (PTG) (n = 359)	0.05	0.62	0.37	1.05	0.074

Adjusted for age, gender, nationality

A parent-reported threat of someone in the family getting COVID-19 or the child getting COVID-19 was associated with a higher likelihood of being in the “High-Risk” cluster. Children whose parents reported the threat of someone in the family dying due to COVID-19 had an approximately 10 times higher risk of being in the “High–Risk” Cluster. Children of parents who had to borrow money were approximately eight times more likely to be in the “High-Risk” cluster. Although not significant, parent-reported posttraumatic growth (PTG) in the child was associated with a 0.62 times lower likelihood of being in the “High-Risk” cluster. PTG was a significant predictor of cluster grouping, as it was associated with a 0.59 times lower risk of being in the “High-Risk” cluster when the logistic regression was not adjusted for age, gender and nationality (p 0.043).

## Discussion

### Summary of findings

We identified a group of children aged 7 to 13 years reporting clinically relevant trauma symptoms and COVID-19-related threats. This group of children is particularly vulnerable to the effects of the pandemic. Parents’ reports of trauma could be used to identify this high-risk group of children.

Approximately 25% of children in the general population had moderate to clinically relevant trauma symptom levels (CATS score > 15 or > 21, respectively) and had parents who reported a high level of trauma symptoms in their children (“High-Risk” Cluster). Although we found some parents’ characteristics that were associated with being in the high-risk group of trauma symptoms, such as financial and job difficulties, there seem to be other factors and mechanisms that exacerbate the experiences and cause a high level of stress response in those children.

After identifying these children, it is important to inform the parents that the children have a high level of trauma that needs to be treated and explained. The high level of trauma was most pronounced in older children (mean age 10.07, SD 1.75).

### Comparison with other studies

To the best of our knowledge, this is the first study that has examined child-reported trauma symptoms during the COVID-19 pandemic. The only available study on PTSD among children during the COVID-19 pandemic was relied on parent reports; that study reported that the prevalence of PTSD among Chinese children was 20.7% [31]. A prospective study of trauma among adolescents during the COVID-19 pandemic found that 20% of adolescents exhibited moderate to clinical levels of psychological trauma [28]. Epidemiological studies have demonstrated that approximately 8–12% of people who experience a traumatic event, such as those caused by a disaster, develop PTSD [19]. In the context of previous pandemics, a study on families who were quarantined due to SARS or the H1N1 influenza virus and based on parental reports found PTSD in 30% of the confined children and in 25% of the parents [51]. A study on home-quarantined youth in China during the first month of the COVID-19 outbreak found that 12.8% of the participants had traumatic stress levels consistent with PTSD [30].

### Prediction of the trauma high-risk cluster

We were able to predict the high-risk cluster with the parent-reported CATS total trauma scale explaining approximately 30% of the variance. Our findings of more frequent financial and job problems in the “High-Risk” cluster than in the “Non-Risk” cluster are also consistent with studies that found that stressful life events and a low socioeconomic status generally increase the risk of reporting mental health problems in children and adolescents aged between 7 and 17 years [45]. Factors related to working from home, such as financial stability, job security, professional autonomy, and schedule flexibility, may buffer parents against psychological distress and negative parenting behaviors [42, 59]. The burden of COVID-19 has not been equal, and families who are socioeconomically disadvantaged have found themselves at greater risk for parent‒child conflict and poor mental health outcomes [11]. Financial stress is known to deleteriously deplete the cognitive, social, and emotional resources available for coping with other life stressors [57].

The majority of children and adolescents exposed to traumatic events develop short-term psychological distress [14]; however, in some children—particularly in those living in families facing a prolonged complex and stressful situation—symptoms do not remit spontaneously and instead become clinically significant, persistent and impairing [51].

There is a growing amount of neuroscientific evidence documenting that early adverse childhood experiences, including prenatal stress and stress throughout childhood, have marked and long-term effects on the development of neurobiological systems (i.e., fronto-limbic circuitry), thereby ‘programming’ subsequent increased stress reactivity and weaker emotion regulation [20]. This altered neurobiological response to stress may confer vulnerability to the development of chronic trauma and stress-related disorders, such as PTSD, anxiety, mood and attachment disorders, memory and learning problems, and other psychopathological conditions [15].

The COVID-19 pandemic has been described as a ‘perfect storm’ with exposure to known risks and the lack of support affecting the mental health of young individuals and their families [13]. First, prolonged lockdown and severe financial difficulties necessitate changes in family dynamics, which may trigger the use of dysfunctional caregiver coping strategies (e.g., alcohol or substance abuse), family discord, negative parent‒child interactions, intrafamilial violence and child abuse [1, 6, 58]. For young children, unsafe living conditions, in parallel with delays in scheduled health care visits and developmental checks, the suspension or interruption of interventions for developmental delays (e.g., language), lost access to child care and early education programs, the disruption of support offered by social services and the loss of supportive social networks, may adversely affect brain development, leading to long-term negative health outcomes [63].

The strong association between parent-reported trauma and child-reported trauma observed herein is inconsistent with previous findings indicating low to moderate agreement between parent- and child-reported trauma exposure and symptoms [18, 52]. Parents seem to underestimate their child’s PTSD symptoms that result from exposure to community violence [8], chronic medical conditions [49] and injury [34, 35]. Consistency between parent and child reported might depend on the context of the trauma exposure. However, there are no data from other studies available on parent and child reports of trauma symptoms during the COVID-19 pandemic. We suggest that the COVID-19 pandemic might affect children and parents in a more similar way than other traumatic events, and thus, children and parents might have similar perceptions of fear and threat to other traumatic events.

### Changes in traumatic symptoms over time on a group level

Comparison of trauma patterns over time by cluster showed that in the “High-Risk” cluster, symptoms increased until reaching a peak in June 2021; then, they began to decrease slightly and remained stable at a very high level (i.e., above the clinically relevant cutoff score of 21). On the other hand, in the “No-Risk” cluster, symptoms remained relatively stable over time at very low levels (a score of approximately 7). A study on adolescents aged 12–18 in Italy conducted during the third lockdown from April 2021 to July 2021 found that almost 18% of participants experienced subthreshold PTSD symptoms, and 41% of the sample indicated that their stress levels had increased since the first lockdown [36]. The literature confirms that stress levels follow a trend, i.e., increasing during the closing phases and decreasing near reopenings [61]. This trend is consistent with the change pattern found in the “No-Risk” cluster, with higher scores in the closing phases of December 2020 and December 2021, but does not explain the high level of symptoms among the “High-Risk” cluster in June 2021 (reopening phase).

Exposure levels (e.g., becoming infected, someone who has been infected in the family) were steadily increasing over time in both clusters, and toward the end of the assessment period in June 2022, almost every child and family member reported having been sick with COVID-19. Another pattern we found was that despite rising exposure levels, trauma symptoms for the “No-Risk” cluster remained stable at a low level, whereas exposure and trauma curves seem to be rising to a similar degree in the “High-Risk” cluster. We therefore suggest that despite the severity of the stressful event being the same for all children, the events themselves were perceived very differently from the children of the two clusters.

With regard to exposure and higher PTSD symptoms, there are inconsistent findings. Some studies showed an association of higher PTSD symptoms with greater exposure levels [25, 53], whereas some studies considered factors such as indirect exposure via COVID-19-related news [9, 29, 37], the impact of “worst” experienced/anticipated events [5] or the subjective severity of a child’s disaster experience [43] to be responsible for higher PTSD symptoms. However, it is important to note that exposure is not identical to stress. Exposure implies a potentially stressful and exhaustive circumstance that may or—as in this group—may not be psychologically taxing [17]. Furthermore, it is known that despite the severe psychological impact of the COVID-19 pandemic, some individuals do not develop high levels of psychological distress [39]. COVID-19 research among children shows that factors such as family cohesion, perceived social support and consistent daily routines can buffer mental health problems and act as significant protective factors against symptomatology [38, 50].

In our study, more Posttraumatic Growth (PTG) occurred in the “No-Risk” Cluster, who reported lower levels of trauma. The PTG model postulates that the severity of the stressful event plays a role in subsequent growth given that fundamental assumptions about the world and oneself become shattered [24].

In general, there is evidence that intermediate levels of posttraumatic growth symptoms are related to higher levels of growth [27]. Nevertheless, in our study, we found that PTG was an indicator of the “normal group”, i.e., the group with the lowest trauma symptoms. Based on the fact that PTG was assessed by parents, it can be assumed that parents facilitated the positive changes they perceived in their children. We suppose that parents of the “No-Risk” children had more opportunities to create and facilitate such a positive environment for their children, while “High-Risk” parents were less likely to do so due to time constraints and socioeconomic disadvantages. Although the facilitation of PTG is not an intervention, it is a legitimate aim in work with children who have experienced a possible traumatic event. Parents might create an appropriate environment to foster PTG [26].

### Clinical implications

As an effect of the COVID-19 epidemic, the total number of psychiatric emergency admissions in 2021 compared to 2020 increased by 40.1% [48]. Currently, health care and school systems are asked to offer help to children with high and subclinical levels of trauma symptoms. When these traumatic symptoms remain, it is likely that these children will develop psychiatric disorders. We recommend adjusting existing intervention programs, particularly for low-income families who have been disproportionately affected by the pandemic but lack access to health care and educational services. Special trauma experts may be engaged to promote children’s posttraumatic growth, which can be a chance to turn the crisis into an opportunity [60]. Public announcements or TV advertisements could help families to reach these settings more easily. For both research and clinical practice, it is important to treat these children in an appropriate way to help alleviate trauma symptoms. Delivering mental health care in nontraditional settings such as schools and primary care may be especially effective for reaching children from low-income households, given that these settings are easier to access and associated with less stigma than mental health specialty clinics [2, 21]. The development and improvement of effective prevention programs and programs to reduce the negative effects of the epidemic on children are needed. As intervention strategies for potentially traumatized children, we suggest educating parents on awareness of their child’s trauma symptoms and needs. Schools may offer programs for mental health, and annual monitoring of mental health in school classes should be in place for the prevention of mental illness.

### Strengths and limitations

The strength of our study was the inclusion of a large sample of children, including a standardized instrument for assessing trauma by child and parent reports, several indicators such pandemic exposure, threat and financial and job difficulties. Given the current paucity of available data with children, these findings provide the first contribution of how the COVID-19 pandemic affected trauma symptoms.

Our study also has limitations. First, self-reported data may not accurately reflect the prevalence of the reported measures. Second, we did not measure the educational background of our participants, but we assume that most of our respondents were children from highly educated and supported backgrounds. Third, our cross-sectional study does not allow us to draw causal conclusions regarding the effects of trauma symptoms, threat and psychopathology in the model. Thus, we are unable to determine whether these aspects preceded the pandemic response or were caused by it. Finally, despite the large sample (n = 752), random selection was not used, and thus, it is impossible to confirm that our sample was fully representative of the population being studied. It is possible that the title of the study and its description attracted parents and children who were highly sensitive to and worried about the COVID-19 pandemic and those who tended to use social media more regularly.

## Conclusions

Further research is required to determine the association between COVID-19 and posttraumatic stress disorders in children. Findings from our study of North and South Tyrolean children clearly show that there is heterogeneity among children regarding their responses to the COVID-19 pandemic.

We identified the following risk factors for experiencing clinically relevant trauma symptoms among children:

- threat that someone in the family gets sick or dies because of COVID-19,
- child being exposed to a family member in hospital or to the death of a family member due to COVID-19,
- high CATS-trauma score levels and CBCL internalizing symptoms (anxiety, depression),
- posttraumatic growth, and
- parents having to borrow money from the bank due to COVID-19.

We have identified risk factors that could lead to clinically relevant trauma symptoms across multiple system levels, including individual biological, psychological, relational, sociocultural, institutional and ecological mechanisms [55]. The findings showed that a weakening of the social system (e.g., parents had to borrow money from the bank, no perception or facilitation of posttraumatic growth) and existing psychological problems of the child (i.e., a weakening of the psychological system) increased the vulnerability of children. Our data also indicate that nationality—North or South Tyrolean—also had an influence on the manifestation of trauma symptoms. To put it cautiously, this finding could indicate that the natural environment (e.g., little access to nature) and the built environment (e.g., how many resources a community can provide) both influence the impact of the COVID-19 pandemic on children's mental health. Future research is required to examine the interconnectedness of multiple systems that could possibly weaken or strengthen a child [32].

## Data Availability

The raw data supporting the conclusions of this article will be made available by the authors without undue reservation.

## Abbreviations

ANOVA: One-way analysis of variance
CATS: Child and Adolescent Trauma Scale Questionnaire
CBCL: Child Behavior Check List
CI: Confidence interval
COVID-19: Coronavirus disease 2019
DSM: Diagnostic and Statistical Manual of Mental Disorders
F: F test in ANOVA
FAMD: Factor analysis of mixed data
M: Mean
P: P value
OR: Odds ratio
PTSD: Posttraumatic stress disorder
PTG: Posttraumatic growth
SD: Standard deviation
*χ*^2^: Chi^2^ test
